# Delayed positive COVID19 nasopharyngeal test, a case study with clinical and pathological correlation

**DOI:** 10.1186/s12890-021-01643-y

**Published:** 2021-08-31

**Authors:** Lei Zhang, Syam P. Vunnamadala, Shigeo Yagi, Riffat Meraj, Michele Carbone

**Affiliations:** 1 Pathology Associates of Anaheim, Anaheim, CA USA; 2 Anaheim Regional Medical Center, Anaheim, CA USA; 3 Doctors Hospital Riverside (Parkview Community Hospital), Riverside, CA USA; 4 California Department of Public Health, Viral and Rickettsial Disease Laboratory, 850 Marina Bay Parkway, Richmond, CA 94804 USA; 5 Thoracic Oncology, Cancer Biology Program, University of Hawai’i Cancer Center, Honolulu, USA

**Keywords:** COVID19, Asthma, COPD, Nasopharyngeal swab, BAL, Corticosteroid

## Abstract

**Background:**

There are various reasons for delayed positive nasopharyngeal PCR tests for coronavirus disease 2019 (COVID19) in not only asymptomatic but also severely diseased patients. The pathophysiological attributes are not known. We explore this possibility through a case report.

**Case presentation:**

A 64-year-old male with history of pulmonary fungal infection, asthma and chronic pulmonary obstructive disease (COPD), diabetes, coronary artery disease presented with shortness of breath, fever and chest image of ground opacity, reticular interstitial thickening, highly suspicious for COVID19. However, nasopharyngeal swab tests were discordantly negative for four times in two weeks, and IgG antibody for COVID19 was also negative. However, serum IgE level was elevated. No other pathogens are identified. His symptoms deteriorated despite corticosteroid, antibiotics and bronchodilator treatment. Bronchoalveolar lavage (BAL) and open lung wedge biopsy were performed for etiology diagnosis. They demonstrated COVID19 viral RNA positive fibrosing organizing pneumonia with respiratory tract damage characterized by suspicious viral cytopathic effect, mixed neutrophilic, lymphoplasmacytic, histiocytic and eosinophilic inflammation and fibrosis besides expected asthma and COPD change. One week later, repeated COVID19 nasopharyngeal tests on day 40 and day 49 became positive.

**Conclusion:**

Our case and literature review indicate that allergic asthma and associated high IgE level together with corticosteroid inhalation might contribute to the delayed positive nasopharyngeal swab in upper airway; COPD related chronic airways obstruction and the addition of fibrosis induced ventilator dependence and poor prognosis in COVID19 pneumonia, and should be therapeutically targeted besides antiviral therapy.

## Background

Since severe acute respiratory syndrome coronavirus (SARS-CoV-2) was isolated in Wuhan, China at end of year 2019, coronavirus disease 2019 (COVID19) has become a global pandemic [1]. In united states, the confirmed COVID 19 cases have surged over 38 million, accounting for 15–20% of the entire world counts. Reverse transcriptase polymerase chain reaction (RT-PCR) is the most commonly used laboratory test for SARS-CoV-2. When performed on upper respiratory swab samples, its sensitivity is reported to be in the range of 60–70% [1, 2]. A considerable proportion of COVID-19 patients may have an initial negative RT-PCR result. Besides the performance characteristic of assays, time of sampling and source of specimen significantly contribute to the “false” negative results [1, 3]. We present a case to discuss one possible pathophysiological explanation, which has not been reported before.

## Case presentation

### Clinical findings

A 64-year-old Asian gentleman was admitted for fever (101.6F), worsening cough and shortness of breath for 10 days. He is a non-smoker and has a remote history of fungal infection of lung over 20 years ago while being a farmer. For the past 3–4 years, he has been taken care in our hospital for persistent and exacerbated asthma, chronic obstructive pulmonary disease (COPD), diabetes, coronary heart disease and congestive heart disease. His home medications for asthma and COPD are albuterol sulfate HFA − 1.25 mg/3 ml, PRN Q4H, Duoneb (a combination of albuterol and Ipratropium Bromide), Budesonid (Pulmicort, cortisone like medicine, NEB 0.5 mg/2 ml) inhalation. During previous asthma exacerbation 13 months ago, methylprednisone injection 40 mg IVP, Q8H and antibiotics Amoxicillin/clavulanic acid were used additionally. During his COPD exacerbation 2 years ago, PrednioSONE TAB—20MG, 40MG PO, daily was also given. In current attack, dexamethasone 10 mg per day, Duoneb and empirical antibiotics azithromycin were initiated in the outpatient setting before admission. The COVID19 nasopharyngeal RT-PCR test performed in doctor's office outside hospital on day 5 of symptom onset was negative.

On admission, his BMI was 21.10 kg/m^2^. His arterial blood gas oxygen was 74 mmHg (normal 75–100) with saturation 96.1% (normal 92–98.5%), and the partial pressure of carbon dioxide pCO2 was 32 mmHg (normal 35–45), pH was 7.48.

The laboratory work showed high white cell count up to 22 × 10^3^/ul with predominant neutrophils (neutrophils 79% and 17 × 10^3^/ul, and the eosinophils accounting for 0–3%), moderate anemia (8–10 g/dl), and reactive thrombocytosis (500–650 × 10^3^/ul). Immunoglobulin E was high (875, normal <  = 114). D-dimer was slightly elevated (2.73 ug/ml, reference < 0.5), as well as N-terminal prohormone of brain natriuretic peptide (NT-PROBNP 1270 pg/ml, reference 0–900). Lactic acid was high 4.6 nmol/L (0.7–1.9). C-reactive protein was also elevated. Ferritin and procalcitonin were within normal limit. The extensive antigen, antibody and culture workups for common infectious disease pathogens such as tuberculosis, hepatitis, HIV, bacteria (including Streptococcus, Legionella etc.), fungi (including coccidioidomycosis, Cryptococcus etc.) were all negative. There was no autoantibody (antinuclear antibody, anti-neutrophil cytoplasmic antibody) either. The repeated nasopharyngeal tests on day 10, 13 and 14 of symptom onset performed in hospital after initial negative result on day 5 were all negative, and IgG antibody for COVID19 on day 16 was also negative.

**Fig. 1:**
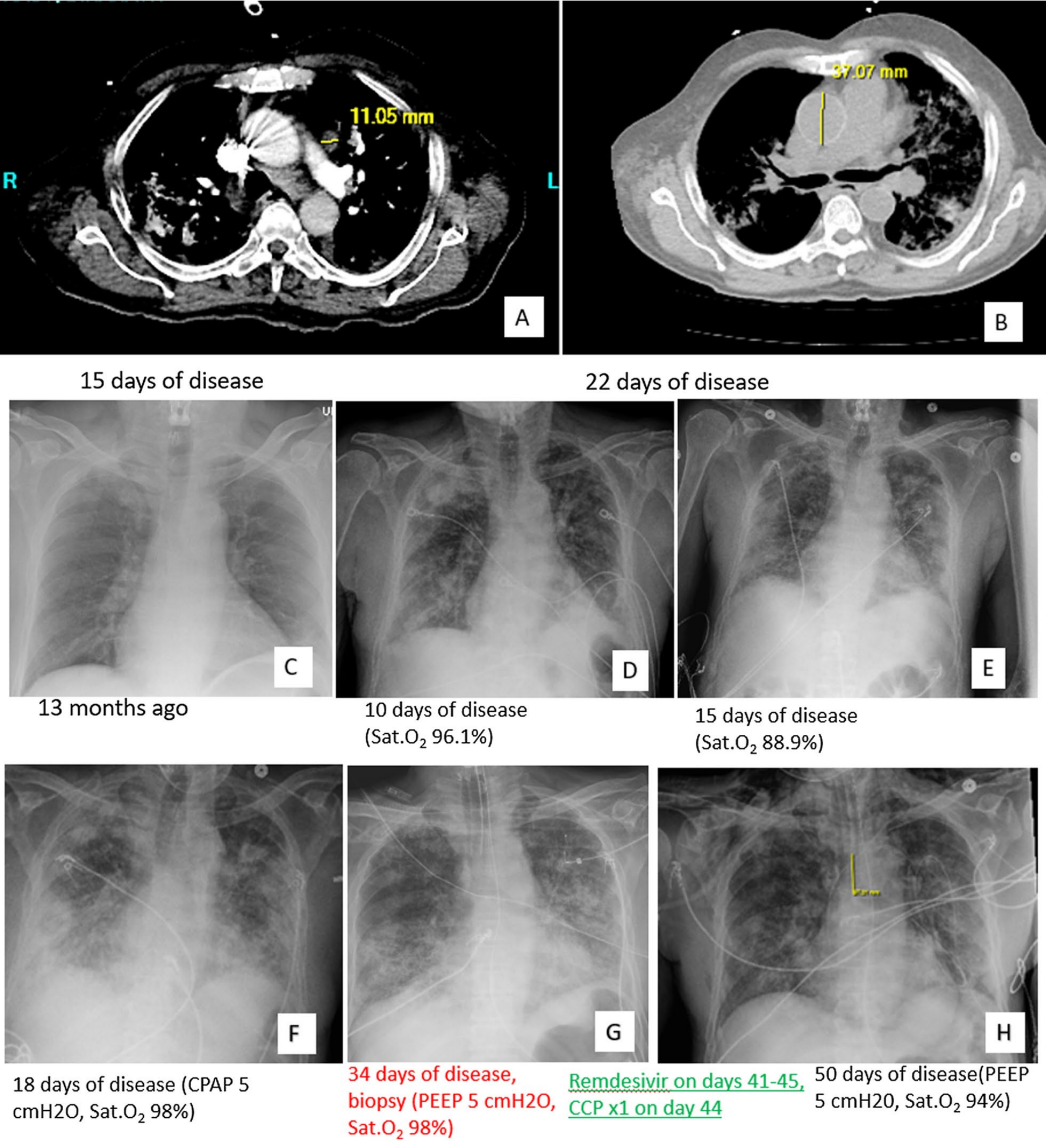
Chest CT and X-ray images. The upper panel (**a** and **b**) shows chest CT and the mid and lower panels (**c**–**h**) show chest X-ray. The images of chest CT on day 15 (**a**) and 22 (**b**) show patchy bilateral lung pulmonary infiltrates with predominant ground-glass components suspicious for pneumonia including COVID19. The new onset disease is mostly peripherally located (**d**–**h**) compared to previous asthma exaggeration 13 months ago (**c**) (mid and lower panel, chest X-ray). The disease was initially stabilized with corticosteroid, antibiotics (including azithromycin) and bronchodilator treatment and patient was not intubated until day 15 (**d** and **e**). The disease deteriorated on day 18 (**f**) and the hypoxemia required ventilation and diagnostic procedure of bronchoalveolar lavage and lung wedge biopsy (**g**). After the confirmation of COVID19, remdesivir, COVID19 convalescent plasma and continuous corticosteroid as well as bronchodilator led to slightly dissolved lung opacity (**h**)

The chest CT and X-ray showed peripherally based ground-glass opacities and consolidation, in contrast with the more centrally located shadings during asthma acceleration 13 months ago (Fig. 1). This is an established imaging manifestation of active COVID-19 infection, but remains nonspecific, as other infections and airspace diseases have a similar appearance. No pulmonary embolism was identified by pulmonary CT.

He was precautiously isolated because of the uncertainty for COVID19. He was managed by empirical antibiotics, inhaled corticosteroid and bronchodilator as well as systemic steroids, besides aspirin, diuresis and diabetic medicines. The major medications addressing the lung disease are:

- Antibiotics: azithromycin (500 mg oral daily, days 5 – 14), ceftriaxone (1 gm, iv, days 9–15), Zosyn (3.375 gm, days 15–19), cefepime (1gm, days 21–31), fluconazole (200 mg, days 22–33), meropenem (1 gm, days 36–40), vancomycin (750–1000 mg, days 36–40).

- Corticosteroid and bronchodilator inhalation medicines for asthma and COPD: Advair (Flutica-Salmet, which is a steroid and bronchodilator combination, MDI 250-50 MCG/puff, days 11–25), Budesonide (Pulmicort, cortisone like medicine, 0.5 mg × 2 per day inhalation, days 16–50), Duoneb (Ipratr-albut, which is a bronchodilator containing beta adrenergic and anticholinergic medicines, 0.5–3 mg/3 ml, Q4H, days 5–50).

-Systemic steroid: dexamethasone (10 mg on days 5–10, 6 mg on days 11–37), methylPRED SUC (Solu-MEDROL, 40 mg, days 16–20, 24–33 and 37–50), and Prednisone (10 mg, days 33–37).

His disease was stabilized initially (Fig. 1e). On day 18, his symptoms deteriorated with Sat.O_2_ dropped from 74 to 54 mmHg. The chest X-ray showed increased bilateral lung opacity (Fig. 1f). The aforementioned medications including antifungal, antibacterial, bronchodilator, inhaled and systemic steroids were no longer helpful improving symptoms (Fig. 1g).

At this time, bronchoalveolar lavage (BAL) was performed to identify an infectious etiology and open lung wedge biopsy was due to rule out interstitial lung disease.

### Pathological findings

**Fig. 2:**
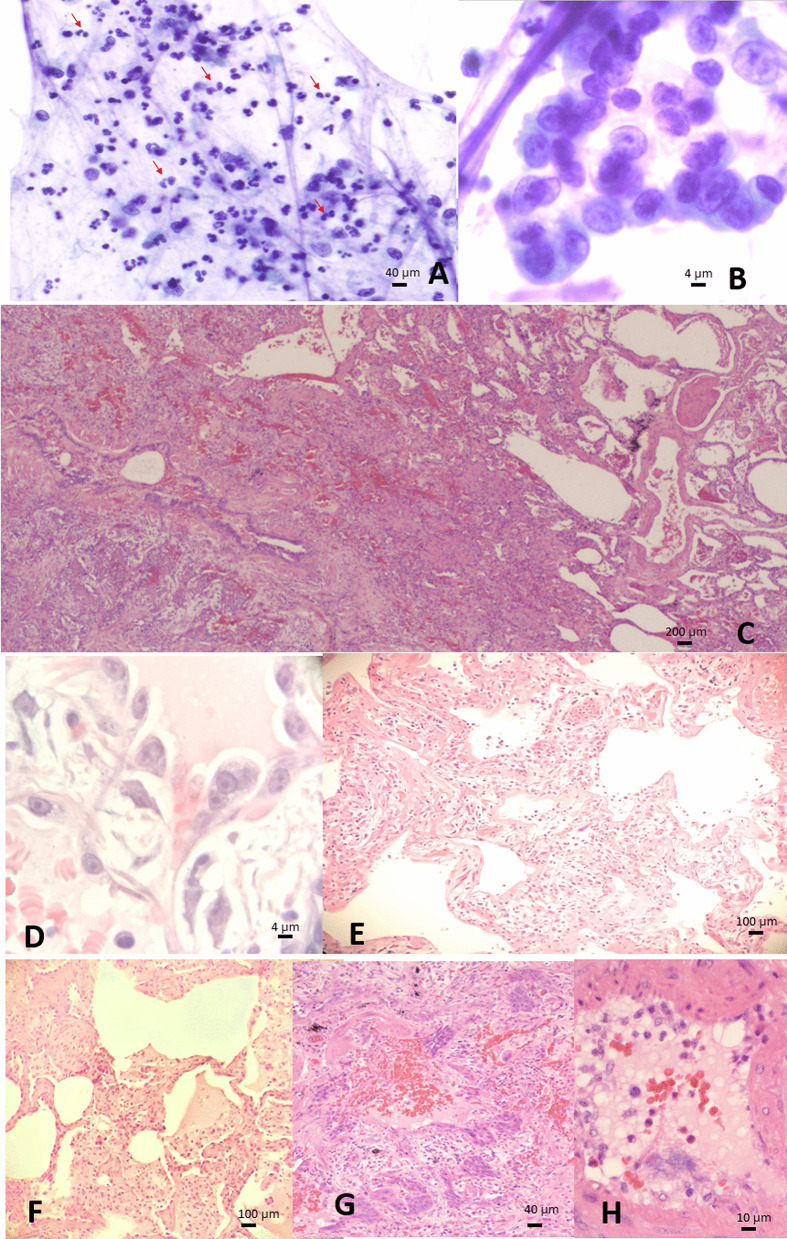
Pathology findings from bronchoalveolar lavage and lung wedge biopsy. Bronchoalveolar lavage showing (**a**) neutrophilic predominant inflammation mixed with some eosinophils (red arrow, compatible with previous history of asthma), and (**b**) reactive pneumocytes with suspicious viral cytopathic effect; (**c**–**h**) lung wedge biopsy revealing asthma change with thickened bronchiolar wall (**c**, left) and mucus plug (**c**, upper right), and COPD which is characterized by emphysema with dilated alveoli distal to bronchiole without fibrosis (**c**, right) and squamous metaplasia reparative to chronic damage (**c**, left lower), as well as newly onset active pneumonia with alveolar and interstitial fibrosis (**c**, mid), type II pneumocytes with possible viral cytopathic effect (**d**), acute pneumonia with alveolar damage (**e**), alveolar edematous exudate, intraalveolar fibrosis (**f**), rare syncytial cells amid squamous metaplasia (**g**), and endothelial injury (**h**). Pictures are taken using Olympus Microscope Model BX45TF, Olympus camera Model DP71 and Olympus CellSens software at a resolution of 72dpi, and processed in adobe photoshop CS5.1 at a resolution of 300 dpi

The bronchoalveolar lavage showed some pneumocytes with acinar arrangement showing enlarged nuclei, prominent nucleoli, open chromatin and mild accentuation of chromatin to nuclear membrane (Fig. 2b) in a background of neutrophilic histiocytic inflammation and occasional eosinophils (Fig. 2a).

The left lingula lung wedge biopsy (Fig. 2c) revealed central bronchiole with thickened smooth muscle wall, and mucus plug, findings consistent with known history of asthma. The presence of central squamous metaplasia and peripheral emphysema indicates chronic airway damage and obstruction suggesting COPD from repeated episodes of asthma. On top of these, there is an active airway centered lesion causing 40–50% alveolar collapse, characterized by fibrosing organizing pneumonia with alveolar damage (Fig. 2e), intraalveolar edematous exudates, fibrosis from intraalveoli extending to interstitial (Fig. 2f), associated with rare syncytial cells in squamous metaplasia foci (Fig. 2g) and capillary endothelial injury (Fig. 2h) accompanied by mixed inflammation comprising neutrophils, lymphoplasmacytic cells, histiocytes and eosinophils in all the process. Again, similar to cytology, reactive large pneumocytes are seen lining hemorrhagic alveoli, showing prominent nucleoli, open chromatin and nuclear membrane accentuation, suspicious for viral cytopathic effect (Fig. 2d). Cytomegalovirus, adenovirus and herpes virus infection is excluded by immunohistochemical stains.

SARS-CoV-2 was detected from both BAL (Quest Laboratory) and lung wedge biopsy (Viral and Rickettsial Disease Laboratory (VRDL), Department of Public Health, California) by RT-PCR. No respiratory syncytial virus (RSV), influenza A or B, parainfluenza virus or adenovirus is identified by PCR (Quest Laboratory). All three laboratories (nasopharyngeal swab RT-PCR performed by Fulgent Genetics, BAL specimen tested by Quest Laboratory, presence of COVID19 in formalin fixed paraffin-embedded lung wedge biopsy tissue verified by VRDL) use the same primer sets unique to SARS-CoV-2 as designed by Center for disease control and prevention (CDC), US. COVID19 pneumonia is confirmed. The repeated nasopharyngeal RT-PCR tests were positive on day 40 and day 49 of disease.

### Follow up

After the diagnosis of COVID19 pneumonia, patient was treated with Remdesivir (200 mg, day 41–45) and COVID19 convalescent plasma (1 unit, on day 44) besides continuous corticosteroid, bronchodilator and supportive management. The granulocytosis subsided to a nadir of 12 K/ul following treatment. The lung opacity started to dissolve a bit slowly (Fig. 1h) but his hypoxemia persisted which still needed ventilation. Gram negative bacteria was isolated from central catheter on day 50. The infected central line was immediately replaced and patient was treated with antibiotics meropenem, transferred to another hospital for continuous care. Patient unfortunately succumbed to septic complications after two months of disease onset.

## Discussion and conclusion

### Different roles of allergic asthma, COPD and others in COVID 19 susceptibility and disease severity

There are conflicting clinical evidences on whether asthma is a susceptible factor for COVID19 infection. However, once allergic and non-allergic asthma is separated, patients with allergic asthma had no statistically significant association with severe COVID-19 symptoms [4, 5].

The COVID19 infection mostly starts from the nose, with virus swept into oropharynx and aspirated into lower lung. For this reason, nasopharynx is a common site for testing COVID19. Angiotensin-converting enzyme 2 (ACE2) and transmembrane serine protease 2 (TMPRSS2) are major cell entry receptors for SARS-CoV-2 infecting human cells. One study revealed lower angiotensin-converting enzyme 2 (ACE2) expression in nasal brushings from those with allergic asthma, along with a progressive decline in ACE2 expression in relation to increasing IgE sensitization [6]. Moreover, it has also been hypothesized that type II inflammatory response cytokines (IL-4, -5 and -13) and accumulation of eosinophils seen in asthma may be protective against COVID-19 [4].

In contrast to asthma, COPD is an established risk factor for COVID19 and associated with disease severity. This is possibly related to elevated ACE2 expression and suboptimal host defense, as well as vascular damage [7]. Among patients with asthma, the expression level of ACE2 and TMPRSS2 in sputum cells in the lower respiratory tract were higher in male sex, African American race and history of diabetes mellitus [8].

Inhaled corticosteroid, the treatment for both asthma and COPD, was associated with lower expression of ACE2 and TMPRSS2 [4, 9]. Corticosteroid inhalation has been shown to suppress coronavirus replication, cytokine production and decrease both ACE2 and TMPRSS2 gene expression in asthma patients [4].

### Delayed positive COVID19 test likely related to asthma, elevated IgE and corticosteroid inhalation

In our case, four nasopharyngeal tests in two weeks of symptom onset were negative for COVID19. The possibility of false omission rate for four negative nasopharyngeal tests in a setting of COVID 19 prevalence of 15–20% is 0.01–0.03, according to a Bayesian model [10]. However, the negative nasopharyngeal results were discordant with the image findings of the lung.

The viral load seems to follow a norm distribution for most recovered COVID19 disease, with a diagnostic window typically between -2 and 18 days of symptom onset for PCR test. The virus is usually not detectable after 20 days of disease by nasopharyngeal test [3]. The serology test starts to be positive after 7 days [3]. In our case, the nasopharyngeal PCR tests were negative on days 5, 10, 13 and 14. However, it was positive on day 40 and 49 after symptom onset. COVID19 IgG antibody was negative on day 16. All those indicate that there is a long latency of virus infection in the upper respiratory tract in this patient.

Patient history of asthma, elevated IgE and corticosteroid inhalation might be associated with modified low level of ACE2 receptor and make virus not detected at nasopharyngeal site initially [4, 6]. On the other hand, the SARS-CoV-2 receptors are elevated in situations of COPD, as well as diabetes and male gender [7, 8]. This overexpression might be more prominent in lower lung where COPD manifests pathologically. This may reflect low virus load below detectable range in the upper respiratory tract, while virus was “concealed” in the lower lung and mirrored by peripheral opacity in the images. The SARS-CoV-2 virus was eventually first identified by bronchoalveolar lavage, which has a high sensitivity of 90% for COVID19, compared to 70% for nasopharyngeal and sputum samples [10].

In the pandemic, hospital capacities are overwhelmed and the testing resources were limited especially in the beginning of disease [1]. Recognition of differential susceptibility in association with various preexistent diseases and apply of testing strategy would prevent erroneously ruling out COVID-19 based on false-negative diagnostic tests. BAL has the highest reported sensitivity but may aerosolize infectious particles. The sensitivity of sputum obtained from intubated patients by deep tracheal suctioning through a closed ventilator circuit has not been reported, but it may present a practical alternative to BAL [10].

### Pathology, image and clinical correlation

The viral cytopathic effects of prominent nucleoli, open nuclei with peripheral chromatin margination, and rare syncytial cells have been reported to be seen in COVID19 infection. Although the cytology features are highly suggestive of COVID19, it is nonspecific. This is the same thing with radiographic images. Peripherally based consolidated opacities and diffuse patchy ground-glass opacities are an established imaging manifestation of active COVID-19 infection, but other infections/airspace diseases could have a similar appearance. The definitive diagnosis currently relies on identification of SARS-CoV-2 RNA sequence.

The onset of COVID19 pneumonia in this patient is associated with granulocytosis in the peripheral blood and neutrophilic extracellular traps in the lung in the absence of other infectious etiology. We did not observe hemophagocytosis in our specimen. Granulocytosis could be due to a shift of neutrophils from the marginated to the circulating pool after corticosteroid treatment. However, the leukocytosis subsided partially after antiviral treatment with Remdesivir and convalescent plasma while patient still being continuously under corticosteroid treatment. This suggests that granulocytosis could be a host reaction to active SARS-CoV-2 virus infection in a subset of patients, probably with comorbidity of COPD. One report has linked the aberrant neutrophilic extracellular traps, so-called “NETs”, to the presence of organ damage in alveolar parenchyma and airways [11]. Peripheral granulocytosis is also associated with poor prognosis in COVID19 disease [11]. The role of “NET” in COVID-19 needs further study.

The lung wedge biopsy on day 34 of disease onset has demonstrated a triplet findings of respiratory cell injury, vascular damage and fibrosis, similar to previously reported COVID19 lung pathology [12]. However, the lung wedge biopsy in our case showed numerous neutrophilic infiltration, which has only been reported in a couple of autopsy cases [11]. This is in contrast with the widely described lymphoplasmacytic cells and macrophages infiltration pattern associated with COVID19 pneumonia [12]. Of note, in the systemic review, which analyzed 198 pathology cases [12], only four patients had COPD and one patient had asthma; none of those patients has a combination of asthma and COPD and the blood IgE level was also unknown.

In the lung wedge biopsy, endothelial cells sloughed off the vascular wall and the small vessels are leaky causing hemorrhage in the alveoli. The endothelial damage might explain slightly elevated D-dimer. No significant perivascular inflammation or vasculitis is identified. There is no significant hyaline membrane seen in the alveoli, a sign of early stage alveolar damage. Rather, there is repair to alveolar damage including squamous metaplasia (related to both current infection and long history of COPD) and evolving fibrosis, which typically occur after 28 days of disease [12]. The pathological findings of fibrosing organizing pneumonia attests the chronological timeline of infection, indicating that active SARS-CoV-2 replication in the peripheral lung occurred before the four negative nasopharyngeal tests. Those fibrosing repair changes have increased the volume of nonaerated lung, making the patient ventilator dependent. Treatment aiding in dissolving the fibrosis could help long survival [1].

## Conclusion

Recognition of differential susceptibility in association with various preexistent diseases can help prevent erroneously ruling out COVID-19 based on false-negative diagnostic tests. Our case and literature review indicate that: (1) Allergic asthma and associated high IgE level together with corticosteroid inhalation might contribute to the delayed positive nasopharyngeal swab in upper airway. (2) COPD related chronic airways obstruction and the addition of fibrosis induced ventilator dependence and poor prognosis in COVID19 pneumonia, and should be therapeutically targeted besides antiviral therapy.

## Data Availability

All data generated or analyzed during this study are included in this article. All data and materials are available for sharing if needed. Please contact corresponding author.

## Abbreviations

COVID19: Coronavirus disease 2019
SARS-CoV-2: Severe acute respiratory syndrome coronavirus 2
RT-PCR: Reverse transcriptase polymerase chain reaction
COPD: Chronic pulmonary obstructive disease
CT: Computed tomography
BAL: Bronchoalveolar lavage
CPAP: Continuous positive airway pressure
PEEP: Positive end-expiratory pressure
ACE2: Angiotensin-converting enzyme 2
TMPRSS2: Transmembrane serine protease 2

## References

[1] Carbone M, Lednicky J, Xiao SY, Venditti M, Bucci E (2021). Coronavirus 2019 infectious disease epidemic: where we are, what can be done and hope for. J Thorac Oncol..

[2] Ai T, Yang Z, Hou H, Zhan C, Chen C, Li W, Tao Q, Sun Z, Xia L (2020). Correlation of chest CT and RT-PCR testing for coronavirus disease 2019 (COVID-19) in China: a report of 1014 cases. Radiology.

[3] Younes N, Al-Sadeq DW, AL-Jighefee H, Younes S, Al-Jamal O, Daas HI, Yassine HM, Nasrallah GK. Challenges in laboratory diagnosis of the novel coronavirus SARS-CoV-2. Viruses. 2020;12:582. 10.3390/v12060582 PMC7354519 32466458

[4] Hughes-Visentin A, Paul ABM (2020). Asthma and COVID-19: what do we know now. Clin Med Insights Circ Respir Pulm Med.

[5] Robinson LB, Fu X, Bassett IV, Triant VA, Foulkes AS, Zhang Y, Camargo CA, Blumenthal KG (2020). COVID-19 severity in hospitalized patients with asthma: a matched cohort study. J Allergy Clin Immunol Pract.

[6] Jackson DJ, Busse WW, Bacharier LB (2020). Association of respiratory allergy, asthma, and expression of the SARS-CoV-2 receptor ACE2. J Allergy Clin Immunol.

[7] Higham A, Mathioudakis A, Vestbo J (2020). COVID-19 and COPD: a narrative review of the basic science and clinical outcomes. Eur Respir Rev.

[8] Peters MC, Sajuthi S, Deford P (2020). COVID-19-related genes in sputum cells in asthma: relationship to demographic features and corticosteroids. Am J Respir Crit Care Med.

[9] Janson C (2020). Treatment with inhaled corticosteroids in chronic obstructive pulmonary disease. J Thorac Dis.

[10] Raschke RA, Curry SC, Glenn T, Gutierrez F, Iyengar S (2020). A Bayesian analysis of strategies to rule out coronavirus disease 2019 (COVID-19) using reverse transcriptase-polymerase chain reaction. Arch Pathol Lab Med.

[11] Barnes BJ, Adrover JM, Baxter-Stoltzfus A, Borczuk A, CoolsLartigue J, Crawford JM, Daßler-Plenker J, Guerci P, Huynh C, Knight JS, Loda M, Looney MR, McAllister F, Rayes R, Renaud S, Rousseau S, Salvatore S, Schwartz RE, Spicer JD, Yost CC, Weber A, Zuo Y, Egeblad M (2020). Targeting potential drivers of COVID-19: neutrophil extracellular traps. J Exp Med.

[12] Polak SB, Van Gool IC, Cohen D, von der Thüsen JH, van Paassen J (2020). A systematic review of pathological findings in COVID-19: a pathophysiological timeline and possible mechanisms of disease progression. Mod Pathol.

